# MoPSeq-DB: a user-friendly web application for genomic data management and analysis of marine mollusc pathogens

**DOI:** 10.1093/database/baaf080

**Published:** 2025-11-26

**Authors:** Clémentine Battistel, Jean-Christophe Mouren, Benjamin Morga, Camille Pelletier, Lydie Canier, Céline Garcia, Isabelle Arzul, Yaelle Pihan, Laura Leroi, Germain Chevignon, Patrick Guido Durand, Maude Jacquot

**Affiliations:** Ifremer, RBE-ASIM, Station La Tremblade, F-17390 La Tremblade, France; Ifremer, RBE-ASIM, Station La Tremblade, F-17390 La Tremblade, France; Ifremer, RBE-ASIM, Station La Tremblade, F-17390 La Tremblade, France; Ifremer, RBE-ASIM, Station La Tremblade, F-17390 La Tremblade, France; Ifremer, RBE-ASIM, Station La Tremblade, F-17390 La Tremblade, France; Ifremer, RBE-ASIM, Station La Tremblade, F-17390 La Tremblade, France; Ifremer, RBE-ASIM, Station La Tremblade, F-17390 La Tremblade, France; Ifremer, IRSI-SeBiMER, Centre Bretagne, F-29280 Plouzané, France; Ifremer, IRSI-SeBiMER, Centre Bretagne, F-29280 Plouzané, France; Ifremer, RBE-ASIM, Station La Tremblade, F-17390 La Tremblade, France; Ifremer, IRSI-SeBiMER, Centre Bretagne, F-29280 Plouzané, France; Ifremer, RBE-ASIM, Station La Tremblade, F-17390 La Tremblade, France

## Abstract

Sequencing technologies continue to evolve, providing novel opportunities for disease surveillance and control. These advancements are crucial for diagnosing diseases and identifying genetically distinct variants with diverse host reservoir species and geographical distributions. Recent progress in sequencing-based analyses of marine mollusc diseases has been significant, yet challenges remain in data management due to a lack of dedicated tools and databases. To address this, we present MoPSeq-DB (Mollusc Pathogen Sequences DataBase), an open-source web application for managing curated genomic data on mollusc pathogens. Designed for accessibility to non-bioinformaticians, MoPSeq-DB features interactive data visualization and integrated analysis tools. Built with the Python Django framework, it automates common bioinformatics workflows, enabling rapid exploration of sequencing data. The application has minimal hardware requirements, and is easy to install, host, and update. MoPSeq-DB facilitates systematic storage and flexible management of genomic data and metadata, improving data organization for mollusc pathogen research. Although developed with a focus on mollusc pathogens, the platform’s adaptable design makes it a valuable resource for studying a wide range of pathogens.

**Database URL**: https://mopseq-db.ifremer.fr

## Introduction

Sequencing technologies continue to undergo constant evolution, offering new perspectives for pathogen surveillance and epidemiology studies [1]. While numerous organizations and research groups have accumulated substantial amounts of sequencing data, efficient storage, management, and visualization are essential to enable in-depth analysis and meaningful interpretation of results [2]. Additionally, genomic data often exhibit heterogeneity, originating from various sources and formats, adding an extra layer of complexity to the management process. Ensuring open access of genomic data to both scientific communities and the general public is also of major interest, enabling a truly open vision for all global pathogen surveillance [3]. Consequently, establishing robust solutions for genomic data management, backup, and sharing is critical to foster collaboration among researchers, expedite scientific discoveries, and drive significant advancements in areas such as public and animal health.

Regarding human and livestock pathogens, genomic studies are widely spread, and various web platforms have been developed to streamline data access and exploration. Most of these platforms have been developed independently, primarily focusing on well-studied organisms such as *Pseudomonas* spp. [4] or rabies virus [5]. These platforms tend to be tied to the characteristics of a specific dataset and adapting their software to other projects could be challenging. Recently, more user-friendly and flexible genome browsers have emerged (e.g. [6]) but they remain quite complex to use for non-bioinformaticians.

The marine molluscan industry represents a vital economic sector worldwide, constituting >18% of the global aquaculture in weight [7]. Despite the frequent occurrence of disease-induced mortality events associated with the industry, a dedicated platform for managing genomic data of marine mollusc pathogens is yet to exist. Mollusc pathogens encompass a diverse range of organisms, including viruses, bacteria, and protozoan parasites (see here for a non-exhaustive list of mollusc pathogens: https://www.eurl-mollusc.eu/content/download/175233/file/TablePathogens_2025_Portrait.pdf). For instance, *Magallana gigas* (formerly *Crassostrea gigas*), one of the most extensively produced marine mollusc species globally [7], is affected by the virus Ostreid herpesvirus type 1 (OsHV-1), leading to mortality rates of up to 100% of spats and juvenile oysters [8]. Additionally, in Europe, adult market-sized *M. gigas* are also susceptible to bacteria like *Vibrio aestuarianus* [9,10]. Molluscan populations are also vulnerable to protozoan parasites such as *Perkinsus olseni*, known to cause infections in various mollusc species including shellfishes like abalones and clams [11,12].

Controlling and preventing marine mollusc pathogens is a complex challenge, primarily because production occurs in an open environment, rendering disinfection and treatment impractical [13]. Furthermore, the frequent movements of animal products facilitate the dispersal of pathogens [14]. Once a pathogenic agent is introduced and spreads within the environment, its eradication is exceedingly difficult, if not impossible [15]. It is therefore essential to prevent the introduction and spread of associated pathogens. In recent years, reference laboratories and researchers have sporadically adopted sequencing approaches to enhance disease diagnosis and understand epidemiology (e.g. [16–18]). However, these efforts have lacked a cohesive framework for pooling available sequences and employing standardized analysis methods, which is essential for consistency among studies.

The principles of FAIR—Findable, Accessible, Interoperable, and Reusable—data play a crucial role in enhancing the accessibility and usability of scientific data across diverse research domains [19]. By adhering to these principles, researchers ensure that their data are easily discoverable, facilitating knowledge exchange and collaboration among peers. Accessibility ensures that data are available for scrutiny and validation, fostering transparency and reproducibility in research. Interoperability promotes seamless integration of data with other datasets and tools, facilitating comprehensive analysis and enabling new insights to be gained. Reusability allows data to be used for future research, maximizing the value and impact of existing datasets. Embracing FAIR principles is paramount in driving the advancement of scientific knowledge, as it promotes a culture of open data sharing and collaboration, ultimately leading to breakthrough discoveries and meaningful contributions to the scientific community.

To address challenges in mollusc disease surveillance, whilst following these principles, we have developed MoPSeq-DB (Mollusc Pathogen Sequences DataBase), an open-source tool based on the Python web framework Django [20]. MoPSeq-DB addresses the practical gap between raw genomic data and biologically meaningful outputs, particularly for non-model organisms such as mollusc pathogens. It is currently designed to manage and visualize genomic data from organisms across multiple kingdoms (viruses, bacteria, and protozoa), is compatible with all modern browser engines (Firefox, Chrome, and Safari) and enhanced visualization features compared to similar platforms. Its user-friendly interface makes it highly accessible, and although initially dedicated to marine mollusc pathogens, the platform can be readily adapted to suit any other pathogens.

## Materials and methods

**Figure 1.**
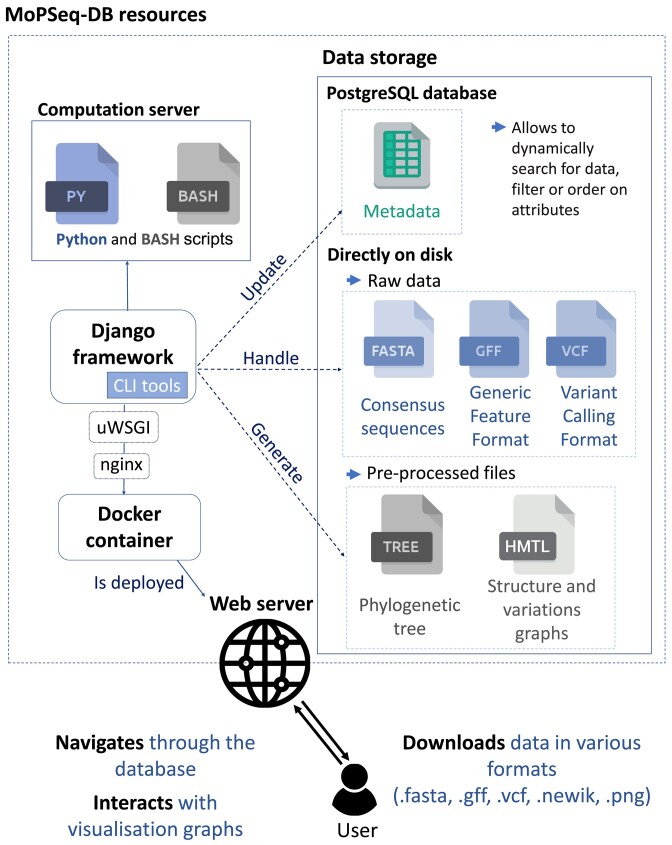
General organization MoPSeq-DB. Each genome is stored in a dedicated storage directory, which contains the assembly file (.fasta), the annotations file (.gff), and detected variations file (.vcf) directly on disk. Simultaneously, all associated metadata is stored in a PostgreSQL database.

MoPSeq-DB is a Python-based web application built on the Django framework v4.2.16 [20]. The general organization of the application is summarized in Fig. 1.

### Data storage

Genome data and associated files are efficiently managed using two references:

- Relational database: A relational database is utilized to handle genome lists and fundamental information (metadata) as described in Table 1. This database serves as a repository for essential information related to each genome, enabling easy retrieval and organization of the data. Additional metadata are saved in the base (related to public database) but not shown to the user on the web interface.
- Disk storage: Each genome is stored in a dedicated storage directory, containing the assembly file (FASTA), annotations file (GFF), and detected variations file (VCF) directly on disk. Pre-processed files (visualizations) for each genome are stored on the disk as well.

**Table 1.** Description of metadata available in MoPSeq-DB

Metadata	Description
Sample name	Name of the sequence. Clicking on it redirects to the genome visualization page of the sample.
Strain	Genetic variant.
Isolate	Population of organisms with minimal genetic mixing.
Host species	Species of the pathogen’s host species.
Collection year	Year the sample was collected.
Collection date	Exact date of sample collection.
Country of origin	Country where the sample was collected.
Location	Locality where the sample was collected.
Latitude/longitude	GPS coordinates of the locality.
Nature of coordinates	Specifies if the coordinates are ‘Verified’ or ‘Approximated’.
Host stage	Developmental stage of the host.
Isolation source	Host tissue from which the pathogen was sequenced.
Pool/individual	Indicates whether the sample originates from a ‘Pool’ of hosts or an ‘Individual’.
Sample conservation method	Method used to preserve the sample prior to sequencing.
Number of contigs	Total number of contigs in the assembly.
Sequencing technology	Platform used for sequencing (e.g. Illumina, Oxford Nanopore, and PacBio).
Sequencer	Name of the sequencing device used.
Sequencing details	Information on paired-end sequencing, read size, etc.
Number of reads	Total number of reads generated in the sequencing run.
Sequence coverage	Average coverage depth of the sequence obtained from mapping.
Sequence size (bp)	Total number of base pairs (bp)/nucleotides in the sequence.
Pathogen load	Quantity of pathogen detected, expressed in copies/µl.
Assembly method	Technique used to assemble the sequence.
Genome type	Indicates whether the genome is complete, non-redundant, or partial.
Structure	Structural regions of the sequence, listed, and separated by “-”.
Name of submitter	Name of the person who submitted or owns the data.
Organization	Institution (e.g. laboratory, university) of the person who submitted or owns the data.
Publication DOI	Hyperlink to the associated publication. Clicking on it redirects to the article.
Notes	Additional information about the sample.
GenBank accession number	Unique GenBank identifier. Clicking on it redirects to the NCBI page.

**Figure 2.**
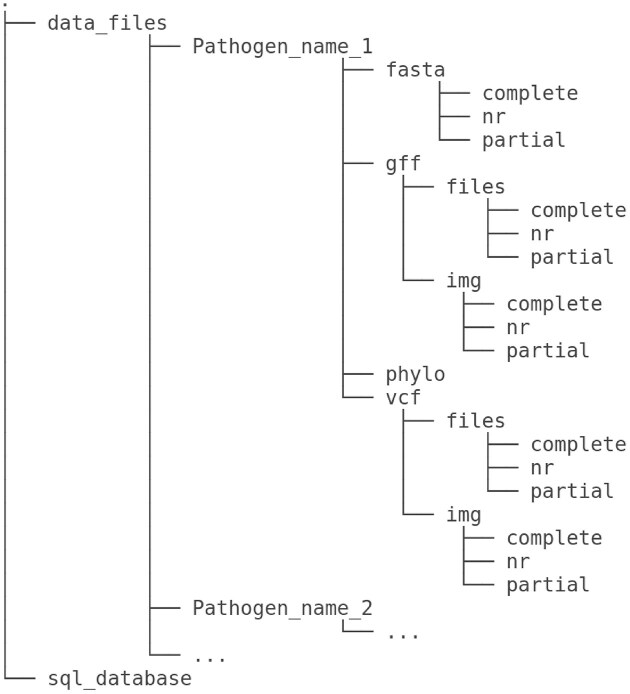
MoPSeq-DB dataset structure. The data repository consists of subfolders corresponding to the pathogens listed in the database, categorized by their attribute ‘Pathogen’ attribute. For each pathogen, the repository contains four main folders (‘fasta’, ‘gff’, ‘phylo’, and ‘vcf’) to store different types of genomic files, further divided into subfolders based on the type of genome. A sample may possess (‘fasta”), as indicated by the ‘genome_type’ attribute in the database. The three folders are as follows: ‘complete’, stores genomic files related to complete genomes; ‘nr’, contains genomic files for non-redundant genomes; and ‘partial’, houses genomic files for partial genomes. In addition, the ‘phylo’ folder contains files related to phylogenetic trees. The ‘gff” and ‘vcf” folders are divided into two subfolders each: ‘files’ and ‘img’. The ‘files’ subfolder is dedicated to storing GFF (General Feature Format) files, while the ‘img” subfolder is used for storing corresponding graphs associated with these GFF files.

This structured data storage hierarchy in MoPSeq-DB (Fig. 2) ensures systematic and efficient management of genomic data, allowing users to easily access and explore the diverse genomic information related to various mollusc pathogens.

### Development vs production environments

During code development cycles, MoPSeq-DB can be used directly on a local desktop computer. To update, test, or run MoPSeq-DB, users can either set up a Python virtual environment (venv) with the required dependencies or use the provided Docker container, which simplifies deployment by eliminating the need for a local venv configuration. By default, MoPSeq-DB web application targets a simple Sqlite3 data bank file v3.39.3 to access data.

However, when turning to production, it is highly recommended to run a dedicated and separated relational database server (e.g. PostgresSQL v2.9.6 [21]). In all cases, MopSeq-DB does not rely on the basic Django web server to run the application. Instead, MoPSeq-DB relies upon uWSGI v2.0.21 (available at: https://github.com/unbit/uwsgi) and NGINX v1.18.0 (available at: https://www.nginx.com/) professional software layers to provide a production class application and for load balancing, allowing it to scale with demand.

### Generation of interactive graphs

To provide interactive visualization graphs, taking into account the various constraints imposed by the different biological models, specific scripts were developed for each kingdom, using a model organism as a basis (OsHV-1 for viruses, *Vibrio aestuarianus* for bacteria, and *Perkinsus olseni* for protozoa). These scripts were designed to be sufficiently generic to be applied to other organisms from the same kingdom.

#### Genomic structures and annotations

To generate genomic structures and annotations graphs, MoPSeq-DB employs a Python script to break down GFF files into interactive Javascript graphs referencing the information contained within the file.

For viruses, the visualizations of genomic structures are generated using the DNAFeaturesViewer package v3.1.1 [22]. The Javascript graphs are dynamically included in the corresponding HTML page of the sample, and their visual rendering is achieved using the Bokeh Javascript package v2.4.2 [23]. Each pathogen agent referenced on the platform possesses its own set of parameters, easily definable for new organisms, and carefully chosen to display the most pertinent features specific to that particular pathogen agent.

For bacteria or protozoa, graphs are generated using Gos v0.1.1 [22], a python library designed to create visualization with Gosling grammar v0.9.31 [24]. In instances where genomes are not assembled at the chromosome level, MoPSeq-DB uses RagTag’s scaffold tool v2.1.0 [25] to artificially gather contigs into chromosomes based on a reference. The alignment of the query assembly and the reference genome is performed using minimap2 v2.17 [26]. The size of gaps between contigs is defined according to the alignment (-r option of RagTag v2.1.0) with a minimum size of 100 bp and a maximum size of 100,000 bp. A minimum unique alignment length of 200 pb is defined (-f 200 option), and contigs not mapped on reference chromosomes are grouped into a dedicated ‘ungathered contigs’ pseudochromosome (-C option). Additionally, MoPSeq-DB incorporates commonly used data in bacteria genome studies for visualization. GC content and GC skew along the genome are calculated along the genome using GC-analysis v0.4.5 (available at: https://github.com/tonyyzy/GC_analysis) and SkewIT’s gcskew tool v1.0 [26] with a window size of 1000 bp and a shift of 100 bp by default.

#### Genomic variations

When available, VCF files were collected from public databases. To handle variations in VCF files, MopSeq-DB employs a python script with the Cyvcf2 package v0.30.15 [26]. This script efficiently organizes the variations based on their type (SNP—single nucleotide polymorphism, insertion, deletions, or complex) and position along the genomes. The variations are stored in different matrices, along with a separate matrix referencing the sequencing depth coverage for each variation.

To provide interactive visualization of these variations, the matrices are converted into Javascript graphs using the Bokeh python package v2.4.2 [23]. These graphs are then seamlessly integrated into the corresponding HTML page for the sample, allowing users to visually explore and analyse genomic variations.

However, in cases where the number of variations becomes too large to generate a graph in a timely manner, MoPSeq-DB implements a filtering mechanism. Variations are filtered on their frequency until the number of displayed variations is reduced to a maximum of 10 000. This approach ensures that users can still access and explore the essential variations while maintaining a responsive and efficient web application experience.

#### Phylogenetic tree

The generation of phylogenetic trees in MoPSeq-DB also varies based on the pathogen’s kingdom.

For viruses, a sophisticated pipeline is employed. It begins with a MAFFT alignment v7.310 [26] of genomic sequences, followed by jModeltest v2.1.10 [27, 28] for evolutionary model selection. The phylogenetic tree is then generated using the PHYML v3.3.3 [29] command line, optimized by jModeltest v2.1.10. This pipeline provides accurate and informative phylogenetic trees for viruses, enabling researchers to analyse their evolutionary relationships effectively.

However, aligning whole genomes of bacteria and protozoan parasites using the above pipeline requires substantial computational resources, making it impractical for MoPSeq-DB—particularly because most available assemblies for these microorganisms are fragmented. Therefore, an alternative approach is adopted for these organisms: the alignment-free software, andi v0.12 [30], is used to estimate evolutionary distances 42, and an R [31] script using the ape library v5.7-1 [32] produces a Newick file based on these estimations. This ensures efficient and resource-friendly tree generation. This alignment-free strategy offers a pragmatic solution to the computational challenges posed by large or fragmented genomes, allowing MoPSeq-DB to provide accurate phylogenetic analyses while ensuring a smooth and scalable user experience.

Once the phylogenetic trees are generated, they are integrated into the dedicated pathogen’s phylogenetic tree visualization page, and rendered using the Phylotree Javascript package v1.0.0 [33].

### Maintenance tool

To streamline and simplify interactions within the MoPSeq-DB project environment, a Python-based Command Line Interface (CLI) tool has been developed. This maintenance tool offers a range of functionalities that aid in managing the database and updating metadata efficiently. The main features of the maintenance tool include:

- Metadata update: Users can easily update metadata associated with genomes using a CSV file.
- Addition of new genomes or organisms: The tool allows for the straightforward addition of new genomes or new organisms to the MoPSeq-DB database.
- Graphs and phylogenetic trees generation: Users can generate various graphs as well as phylogenetic trees using the CLI tool.
- File sorting: The maintenance tool also offers the capability to sort all the files stored within the folder architecture of MoPSeq-DB.

Overall, the Python CLI maintenance tool plays a vital role in enhancing the usability and efficiency of MoPSeq-DB, providing with a seamless experience in managing and analysing genomic data related to mollusc pathogens.

### FAIR principles applied to MoPSeq-DB

MoPSeq-DB follows FAIR principles [19] by providing all data and source code through stable links (DOI for data and institutional GitLab portal for source code) and documentation. Regarding data, all pieces of information displayed by the web portal is also available through data servers from Ifremer’s International Oceanographic Data Center, a CoreTrustSeal certified service (information at: https://www.coretrustseal.org), using standard data formats (e.g. SQLite dump for data tables, or FASTA for consensus sequences, etc.). It is worth noting that MoPSeq-DB relies on public data, so links to original sources of information (e.g. EBI-ENA, NCBI) are provided by MoPSeq-DB as well.

## Results

The outcome of our work is a user-friendly platform (available at: https://mopseq-db.ifremer.fr/) that provides a simple yet comprehensive overview of various molluscan pathogen genomes.

### General organisation

MoPSeq-DB allows users to (i) navigate through a comprehensive and curated collection of publicly available mollusc pathogen genomes (virus, bacteria, and protozoan), (ii) access fully interactive views of genome structures, annotations, variants, and phylogenetic trees, and (iii) download data in various formats: Fast-Alignment format (FASTA; .fasta), General Feature Format (GFF; .gff), Variant Call Format (VCF; .vcf), Newick phylogenetic tree, and PNG.

It is worth noting that MoPSeq-DB software design takes care to separate the core application layer (i.e. Django application) and the data provider layer (e.g. files on disk and relational data server, see below). Moreover, MoPSeq-DB is fully designed to benefit from the Continuous Integration/Continuous Delivery (CI/CD) framework included in GitLab and Github. MopSeq-DB is hosted at Ifremer GitLab instance where the application has been fully configured to automatically enable CI (automatic building of the Docker image on new release tag) and CD (automatic deployment on Ifremer web servers of that new release).

### Data retrieval

MoPSeq-DB functions as a downstream repository for mollusc pathogen genomic data collected from principal public repositories for sharing genomes, as GenBank and Assembly databases from the National Center for Biotechnology Information (NCBI) [34] and the European Nucleotide Archive (ENA) [35]. Data are gathered twice a year or on demand. In addition to sequence data, feature annotations and sequence variations are also curated. Metadata is transferred in a standardized format to enhance the value of these genomic data. Metadata collection aims to be as complete as possible and includes up to 51 fields related to sample collection (e.g. location or date), sequencing technologies and assembly methods.

As for now, MoPSeq-DB contains genomic data and metadata for 80 genomes of OsHV-1 (complete or non-redundant, 12 of which are unpublished in public repositories yet), 96 genomes of *Vibrio aestuarianus* (complete or full fragmented, 1 of which are unpublished in public repositories yet), and 5 genomes of *Perkinsus olseni* (full fragmented). However, the platform is continuously evolving, and as mollusc pathogens sequencing progresses, it will be regularly updated and enriched with new data related to the species already present in the DB but also to new pathogens. Data provided through the MoPSeq-DB web interface are also available for direct access and loading through Sextant catalogue, a CoreTrustSeal (https://www.coretrustseal.org/) certified data-providing service of Ifremer here: https://doi.org/10.12770/52134702-4bbd-4c63-af34-9a2cde28e0cc.

### Data sharing

**Figure 3.**
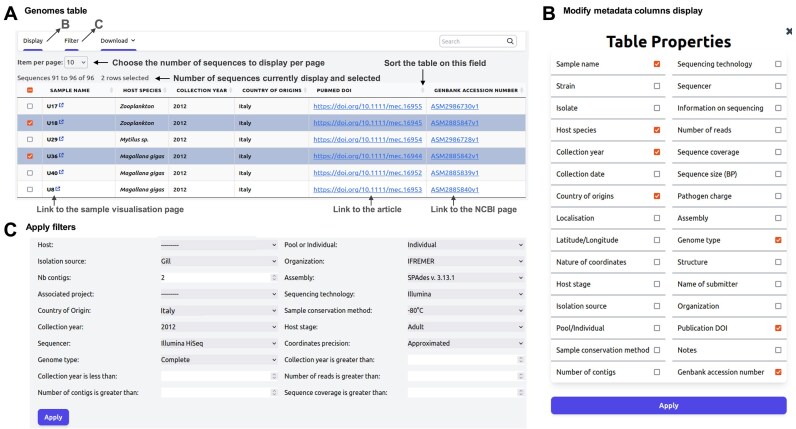
Annotated screenshots of genome tables. (A) Sortable and filterable table. (B) Genome context menu: provides access to additional features. (C) ‘Show columns and filters’ bar: clicking on this bar expands settings to add more metadata columns to the table and apply filters.

Users can access a wealth of information and have the option to download associated genomic files (FASTA, GFF, and VCF) of samples. The platform offers dynamic data searching and filtering functionalities through selectors based on different attributes (Fig. 3). Users can also customize data presentation by ordering, displaying, or hiding specific attributes to tailor downloads according to their needs.

### Interactive graphs

MoPSeq-DB adds value to other public sequence repositories in several ways, particularly by enabling data visualization through interactive graphics. To facilitate seamless data visualization, MoPSeq-DB provides a comprehensive set of data handling scripts. These scripts perform essential pre-computations for generating various processed data files, such as phylogenetic trees and interactive Javascript-based graphs. These pre-processing steps significantly enhance the efficiency of data retrieval and dynamic visualization of genomic information, resulting in an improved overall usability and performance of MoPSeq-DB.

#### Genomic structures and annotations

**Figure 4.**
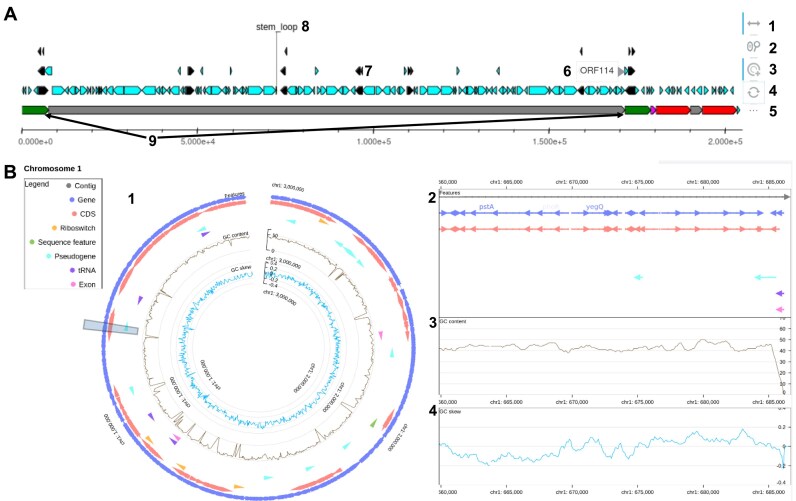
Screenshots of genomic structure and annotation visualizations in MoPSeq-DB. These annotated screenshots illustrate the interactive genomic structure graphs available in MoPSeq-DB, providing users with various interactive options for in-depth exploration of genomic data. (A) For viruses: 1—horizontal movement: navigate along the genome by moving horizontally on the graph; 2—wheel zoom: zoom in and out to inspect specific regions of interest; 3—element selection and isolation: select and isolate specific elements for focused examination of genomic features; 4—revert to base image: reset the graph to its original state; 5—disable labels: remove labels to declutter the visualization; 6—label of a structural region; 7—representation of elements stored in .gff files (CDS, genes …); 8—fixed label for ‘stem_loop’ region; 9—repeated regions are highlighted in the same colour (e.g. green for ‘IRL’). (B) For bacteria and protozoa, one graph is generated per chromosome. Data are visualized on two scales: 1—an overview of the chromosome; 2—an interactive detailed view, allowing horizontal movement, zooming, and access to information about specific elements; 3—GC content; and 4—GC skew along the genome.

Despite being conceptually and technically straightforward, searching for annotations in a set of genomes can be tedious or even impossible for non-programmers. In MoPSeq-DB, two types of graphs are generated to make annotation searches quick and easy: one linear for viruses and the other circular for bacteria and protozoa (Fig. 4), allowing access to additional information as GC content and GC skew for bacteria graphs (Fig. 4B).

As samples may have different sequence files due to varying genome types (circular, linear, number of replication units, complete, non-redundant, or partial), MoPSeq-DB generates a distinct graph for each of these sequence files. Each graph is displayed on the sample visualization page, providing relevant information specific to the corresponding file, one at a time to guarantee a fluid user experience. This approach allows users to explore the genomic information in a structured and manageable manner, enhancing the overall usability and accessibility of MoPSeq-DB.

#### Genomic variations

The graphs depicting genomic structures and variations in MoPSeq-DB are highly interactive, offering several options for users to explore the data effectively. For variation graphs, users can utilize numerous interactivity features, such as saving an image of the graph and toggling the display of dots corresponding to specific types of variations they are specifically interested in (Fig. S1). The different types of variations that can be displayed are single or multiple nucleotide polymorphisms (SNP/MNP), insertions, deletions, or more complex variations (combinations of different types of variations occurring in close proximity). Reference corresponding nucleotides can also be displayed or masked on the graph.

#### Phylogenetic trees visualisation

The phylogenetic trees created for MoPSeq-DB can be accessed both on individual genome pages and on a dedicated page. When viewing a genome page, the tree highlights the specific genome within the phylogenetic tree.

**Figure 5.**
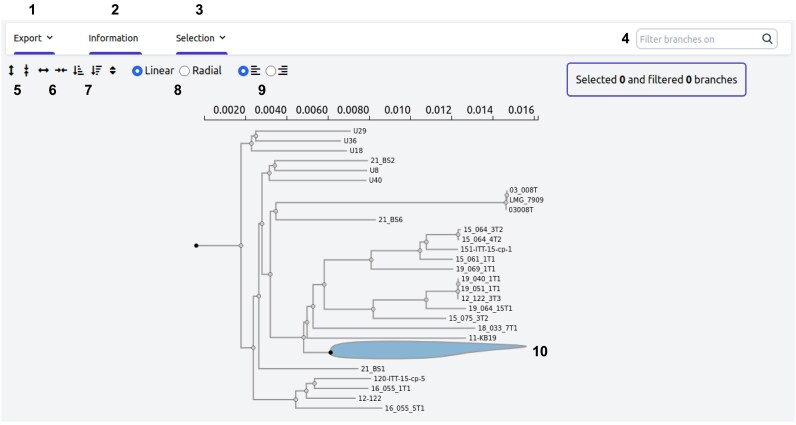
Phylogenetic tree visualization. This annotated screenshot displays the interactive phylogenetic tree available on MoPSeq-DB for *Vibrio aestuarianus*, providing users with several interactive options. 1—Export: save the tree in Newick format or as an image with the current selection; 2—information: access the commands used to generate the tree; 3—selection: select different elements within the tree; 4—search for samples and highlight them directly; 5—enlarge or shrink the tree vertically; 6—enlarge or shrink the tree horizontally; 7—reorganize the tree based on clade size; 8—switch between linear or radial configuration; 9—align sample names or attach them to the tree branches; 10—hide or collapse sub-trees for a cleaner view.

Phylogenetic trees present different interactivity options for the user to better handle data and visualize results. The users can zoom or reorganize the trees with different configurations to better fit their needs. It is also possible to collapse or hide subtrees, select or filter different sequences or groups, or re-root the tree as described in Fig. 5. Downloading the file or a high-quality image of the tree with the current highlighting is also possible.

This enables users to explore and interpret the evolutionary relationships among different pathogen agents, providing valuable insights into their genetic relatedness and evolutionary history. The visualization of phylogenetic trees enhances the understanding of the diversity and evolution of mollusc pathogens, thus contributing to the comprehensive data exploration capabilities of MoPSeq-DB.

To ensure transparency and traceability in phylogenetic tree calculations, our workflows automatically generate a report during the tree-building process. This report details the name and version of each software used, along with the settings applied, and displays this information on the web page.

## Discussion

MoPSeq-DB is, to our knowledge, the first genome browser dedicated to marine mollusc pathogens. In line with FAIR principles [19], it is a valuable and powerful resource that empowers researchers and regulators in the context of mollusc diseases. This platform represents, therefore, a significant milestone in genomic research within the field of mollusc diseases and sets the stage for worldwide collaborative and impactful research.

While general-purpose platforms such as OpenGenomeBrowser [6] offer broad organism coverage and advanced features, they are often tailored for bioinformaticians and require technical expertise. MoPSeq-DB, in contrast, is specifically designed for the marine mollusc pathogen community, offering a user-friendly interface for researchers without advanced bioinformatic skills, laboratories seeking to standardize and share genomic outputs and stakeholders. Unlike curated databases such as the Pseudomonas Genome DB [4], which focus on a single bacterial species, MoPSeq-DB supports multiple pathogen kingdoms (viruses, bacteria, and protozoa) and integrates custom visualization logic for each. Additionally, tools like DNA Features Viewer [22] provide visualization libraries but lack an integrated interface or data management capabilities (Table 2).

**Table 2.** Comparison of MoPSeq-DB with other genomic platforms and tools

Feature	MoPSeq-DB	OpenGenomeBrowser	Pseudomonas Genome DB	DNA Features Viewer
Primary focus	Marine mollusc pathogens	General-purpose, multi-organism datasets	*Pseudomonas aeruginosa* only	Generic visualization library
Self-hostable/portable	Yes (Docker, open source)	Yes (Docker, modular design)	No (centralized hosted platform)	Yes (Python library, local use)
Customizable for other pathogens	Yes (kingdom-specific visualization scripts, and parameterization)	Yes (data-agnostic design)	No	Yes (requires manual configuration)
User interface	Web-based, intuitive, designed for non-specialists	Web-based, feature-rich but more technical	Web-based, expert-oriented	No UI (script-level visualization)
Comparative genomics support	Yes (annotations, variants, metadata, and phylogenetic analysis)	Yes (phylogeny, gene loci comparison, metabolic pathways)	No	No
Kingdom-specific visualisation	Yes (custom rendering for viruses, bacteria, protozoa, with taxon-specific logic)	No (generic genome views only)	No	No
Data updating mechanism	Python CLI for metadata updates, genome additions, and visualization generation	Semi-automated (file system + metadata files)	Manual curation by internal experts	N/A
Scalability/performance	Production-ready stack (uWSGI/NGINX, Docker), supports SQLite3 or PostgreSQL, filtering logic for large datasets	Docker-based deployment, supports SQLite3 or PostgreSQL	Limited to a single curated dataset	Local script performance only
Target user base	Researchers in mollusc diseases, non-specialists researchers, diagnostic labs, managers monitoring pathogen diversity	Bioinformaticians, comparative genomics researchers	Microbiologists and pathogen-specific curators	Developers and computational biologists

One of the strengths of MoPSeq-DB is its adaptability in managing genomic data beyond mollusc diseases. This flexibility opens up opportunities for collaborative research across different fields and enables the platform to cater to a wider scientific community. By providing a centralized and standardized database, MoPSeq-DB fosters consistency in international studies and facilitates curated data sharing among researchers, promoting knowledge exchange, and accelerating scientific progress.

The platform’s user-friendly interface, data management capabilities, and interactive graph visualizations offer a robust framework for researchers and shellfish industry stakeholders to explore genomic data available and gain valuable insights into the genetic characteristics of mollusc pathogens. It automates commonly used bioinformatics workflows, enabling convenient and fast data exploration, particularly for non-bioinformaticians, in an intuitive way. As this platform continues to evolve, it holds the potential to make significant contributions to the global efforts in understanding and controlling the impact of mollusc diseases on the aquaculture industry and marine ecosystems. This involves, in particular, increasing the accessibility and reproducibility of genomic data analysis, by making analysis workflows available from the platform. This ensures that limited computer science training or lack of computer resources is not barriers to genomic analysis, and also allows for better comparison of results [2].

While MoPSeq-DB already contains a substantial amount of genomic data, its potential for growth is considerable. As mollusc pathogen sequencing progresses and new data become available, MoPSeq-DB will play a pivotal role in organizing and providing access to an ever-expanding repository of genomic information. This expansion will not only deepen our understanding of mollusc diseases but also contribute to broader scientific research in pathogen genomics and epidemiology.

Indeed, the comprehensive set of data handling scripts in MoPSeq-DB streamlines data management and updates, ensuring that the platform remains current with the latest genomic information. In the near future, additional tools will be integrated into the platform to facilitate automated requests to major public repositories, primarily GenBank and Assembly databases from the NCBI [34] and ENA [35], for effective genome surveillance. This functionality is similar to what is offered by some other recent genomic sharing platforms (e.g. [36]) and it plays a crucial role in the rapidly evolving field of genomics, where new discoveries are constantly being made. While we believe a human check will still be needed to maintain the quality of data included in our database, this proactive approach will bolster MoPSeq-DB as a dynamic and cutting-edge resource.

The tool has minimal hardware requirements and is easy to install, host, and update. MoPSeq-DB folder structure enforces systematic yet flexible storage of genomic data of mollusc pathogens, including associated metadata. The platform could easily be adapted for use with other pathogens, as it was designed to allow rapid adaptation to genomic data from a wide range of microorganisms. Therefore, it represents a good alternative to other recent high-performance and flexible genome browsers (e.g. [37]). In addition, MoPSeq-DB has been designed with scalability in mind to ensure long-term viability and adaptability. The application relies on production-grade deployment tools such as uWSGI and NGINX, and supports the use of PostgreSQL as a robust relational database system for handling large volumes of genomic metadata. Its architecture separates the application logic from the data layer, enabling horizontal scaling and load balancing if necessary. Furthermore, Docker-based containerization allows for flexible deployment and replication in various hosting environments. A variation filtering mechanism ensures performance and responsiveness even with highly variable genomes, by limiting the number of rendered elements per sample. These design choices enable MoPSeq-DB to efficiently handle large datasets and serve multiple users concurrently without degradation in performance.

Additionally, we plan to extend the scope of the platform to include a system for viewing and integrating spatial data information in a more sophisticated manner, and therefore allowing the joint analysis of epidemiologic and genomic data. In particular, including visualization of phylogeographic results to MoPSeq-DB would be valuable. Through the integration of Nextstrain modules [38], our platform would offer real-time population cartography, providing users with dynamic and interactive insights into the geographic distribution and evolutionary history of mollusc pathogens. More generally, this powerful enhancement could enable mollusc disease researchers, professionals, and stakeholders to explore the intricate patterns of pathogen spread and evolution, shedding light on critical factors influencing mollusc disease transmission and/or emergence.

There are also plans to adapt the database to allow for the addition of other types of data, such as genetic marker sequences (e.g. 18S, multi-locus sequence analysis) currently used in the study of various species of protozoan parasites [39] or for the characterization of *Vibrio* bacteria [40]. These markers correspond to DNA fragments that enable the characterization of the pathogen type at the genus level and even at the species level, but not at the strain or lineage level [41]. Obtaining complete genomes for most protozoan parasites of marine molluscs is complex because (i) most of these organisms are not cultivable, making it difficult to obtain purified genetic material in sufficient quantities for sequencing, and (ii) very little knowledge is available about their genomes (size, number of chromosomes, etc.), making the assembly of raw sequencing sequences highly complicated. For instance, adding marker sequence data for *Marteilia refringens* would involve the incorporation of ∼500 DNA fragments ranging from 300 to 3000 bp from three different markers (18S, IGS, and ITS1).

Throughout this and other future efforts, we aim to continue to provide a high-quality, user-friendly, and powerful resource to facilitate broad access to advanced pathogen surveillance, diagnostics, and mitigation strategies for the mollusc research community and industry actors. Users are encouraged to contact MoPSeq-DB’s maintenance team to request the inclusion of new datasets or features, ensuring the platform continues to evolve and meet the growing needs of its community.

## Data availability

Source code, documentation, docker container, and a test dataset are available at: https://gitlab.ifremer.fr/bioinfo/mopseq-db. Data included in the database can be downloaded either from MoPSeq-DB web application (available at: https://mopseq-db.ifremer.fr) or from the Sextant catalogue (accessible at: https://doi.org/10.12770/52134702-4bbd-4c63-af34-9a2cde28e0cc).

## Ethics

This research project did not require any ethical approval.

## Supplementary Material

baaf080_Supplemental_File
