# Supplementary figures and images for: MoPSeq-DB: a user-friendly web application for genomic data management and analysis of marine mollusc pathogens
Source: Database (Oxford). 2025 Nov 26;2025:baaf080. doi: 10.1093/database/baaf080 (PMC12648391; doi:10.1093/database/baaf080)

## Slide 1
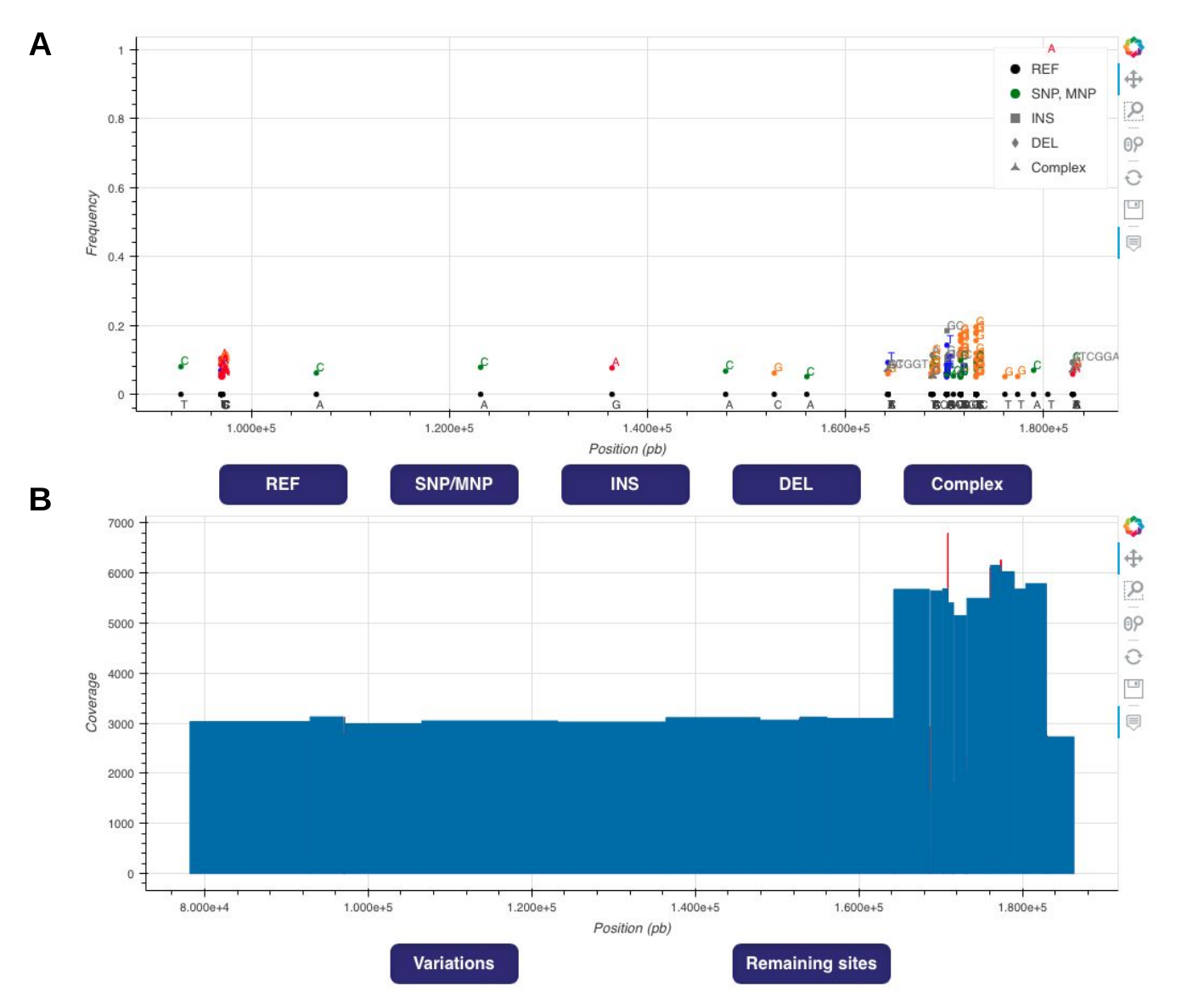

A
B

Supplement: baaf080_Supplemental_File [file baaf080_supplemental_file.zip › FigureS1.pptx]
